# Hierarchical Transparent Back Contacts for Bifacial CdTe PV

**DOI:** 10.1021/acsenergylett.4c00156

**Published:** 2024-03-20

**Authors:** B. Edward Sartor, Teng Zhang, Christopher P. Muzzillo, Chungho Lee, Ryan Muzzio, Yury Gogotsi, Matthew O. Reese, André D. Taylor

**Affiliations:** † New York University, Brooklyn, New York 11201, United States; ‡ National Renewable Energy Lab, Golden, Colorado 80401, United States; § A.J. Drexel Nanomaterials Institute, Drexel University, Philadelphia, Pennsylvania 19104, United States; ∥ First Solar, Santa Clara, California 95050, United States

## Abstract

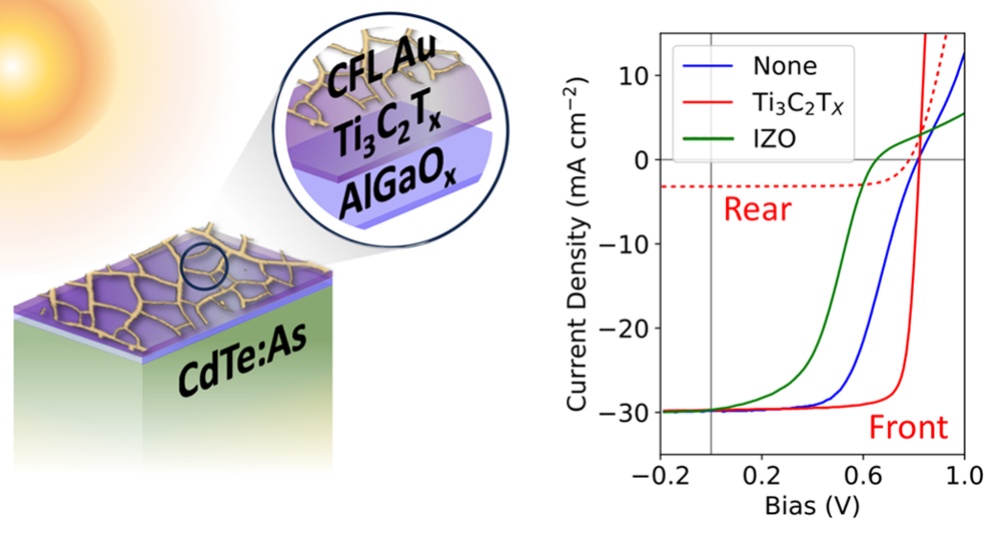

A hierarchical transparent back contact leveraging an AlGaO_*x*_ passivating layer, Ti_3_C_2_T_*x*_ MXene with a high work function, and a transparent cracked film lithography (CFL) templated nanogrid is demonstrated on copper-free cadmium telluride (CdTe) devices. AlGaO_*x*_ improves device open-circuit voltage but reduces the fill factor when using a CFL-templated metal contact. Including a Ti_3_C_2_T_*x*_ interlayer improves the fill factor, lowers detrimental Schottky barriers, and enables metallization with CFL by providing transverse conduction into the nanogrid. The bifacial performance of an AlGaO_*x*_/Ti_3_C_2_T_*x*_/CFL gold contact is evaluated, reaching 19.5% frontside efficiency and 2.8% backside efficiency under 1-sun illumination for a copper-free, group-V doped CdTe device. Under dual illumination, device power generation reached 200 W/m^2^ with 0.1 sun backside illumination.

Bifacial solar deployment reached 8.8 GW_Dc_ in 2019 and is projected to account for 75% of the solar market by 2025.^1^ Efficient bifacial devices have been made using many commercial and precommercial semiconductors including Si, GaAs, and perovskites. For example, silicon modules are available with up to 80% bifaciality, characterized by the ratio of power conversion efficiency (PCE) when illuminated from the frontside over PCE under backside illumination (PCE_Back_). Cadmium telluride (CdTe), the most widely deployed thin-film photovoltaic system on the market with ∼8 GW of global production capacity to date and an expectation of >25 GW of production available by 2027, has yet to demonstrate efficient bifacial contacts, with PCE_Back_ topping out at ∼20% of the record CdTe device efficiency.^2^

Several challenges have precluded the application of bifacial contacts to CdSeTe semiconductors (Figure 1a,b). The back surface of a superstrate CdSeTe deposited with close-space sublimation or vapor transport deposition has a high density of both electronic defects and charged species, resulting in the rapid recombination of carriers generated near the back surface.^3^ In addition, the formation of an ohmic contact with transparent p-type contacts has been elusive. CdSeTe has a high ionization potential of 5.9 eV, resulting in counterproductive band bending that pushes charge carriers toward recombination centers and introduces series resistances in critical cases.^4^ The requirements for the bifacial contact material itself are no less stringent, requiring high transmissivity and conductivity to allow for light ingress through the back surface while maintaining a low series resistance.

**Figure 1:**
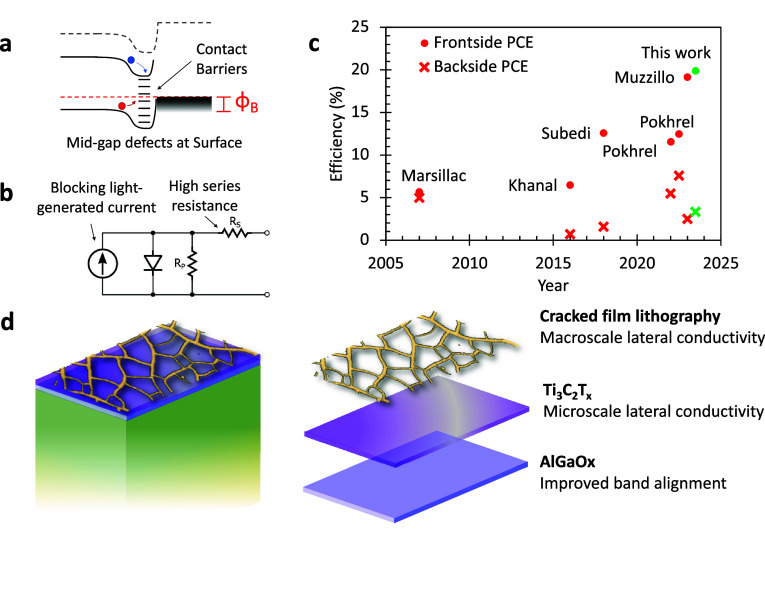
(a) Band diagram at the back of a CdTe device demonstrating how carriers generated near the back surface experience detrimental recombinative events due to the presence of contact barriers and midgap defects near the surface. (b) Circuit diagram indicating key resistances and current-generating elements in a bifacial configuration. (c) Historical bifacial CdTe front and backside photoconversion efficiencies. (d) Hierarchical back contact scheme.

Several bifacial architectures have been pursued (Figure 1c). Marsillac et al. demonstrated ultrathin sputtered semitransparent and bifacial CdTe, with a frontside efficiency of 5.7% and a backside efficiency of 5%, with the high bifaciality ratio attributable to the favorable generation profile of an ultrathin absorber under illumination from either side.^5^ A substrate device designed with a carbon nanotube p-type contact on the glass side resulted in 6.5% frontside efficiency.^6^ Thicker devices have proven more challenging, as generating light farther from the main device junction requires improved lifetimes to allow charge carriers to be extracted. Nanoparticle (NP) solutions have yielded promising results, with a 13% PCE, 12% bifaciality CuS/ZnS contacted device and an 11.5% PCE, 47% bifaciality CuI NP/indium tin oxide (ITO) contacted CdTe device demonstrated.^7,8^ Doped and alloyed metal oxides have provided a wealth of potential candidates, with a CuCrO_*x*_ contact outperforming other oxides with a 12.5% PCE, 60% bifaciality device demonstrated.^9^ Recently, Muzzillo et al. demonstrated a combined CuGaO_*x*_ and templated Au nanogrid contact with 19.2% frontside efficiency and 2.5% backside efficiency.^10^ This hierarchical approach is extended in this work to use a copper-free AlGaO_*x*_ buffer layer, a conductive and semitransparent Ti_3_C_2_T_*x*_ interlayer with a high work function, and a highly conductive patterned gold nanogrid grid to lower series resistance.

Solution-processed AlGaO_*x*_ hole transport layers have a tunable band gap based on the ratios of aluminum and gallium precursors. Evidence for the passivating quality of an aluminum oxide thin film has been previously reported for CdTe surfaces.^11^ Akkuly et al. report an undoped CdS/CdTe AlGaO_*x*_ passivated device with an opaque 45 nm Au contact.^12^ However, the role of AlGaO_*x*_ as a surface passivant has not been comprehensively explored or confirmed, particularly in doped devices. Incorporating GaO_*x*_ into the AlO_*x*_ film increases the conductivity of the thin film through doping with oxygen vacancies, lessening detrimental transverse resistances as carriers pass through the transport layer. However, in-plane series resistances prevent AlGaO_*x*_ from being used as a final contact layer, requiring additional metallization to complete a device. However, in the interest of fabricating a bifacial device with a semitransparent contact, an alternative metallization to bulk Au is necessary.

MXenes, a family of two-dimensional transition-metal carbides, nitrides, and carbonitrides discovered in 2011, have shown potential for CdTe metallization due to their high electrical conductivity, hydrophilic surface, and tunable chemical composition.^13^ MXenes have the general formula M_*n*+1_X_*n*_T_*x*_, where M is an early stage transition metal, X is carbon and/or nitrogen, and T_*x*_ represents surface terminations such as =O, -OH, -F, and -Cl which arise from synthesis and postprocessing conditions. By controlling the transition metal (M) and surface terminations (T_*x*_), one can modulate the electronic and optical properties of metallic MXene.^14,15^ The layered structure enables efficient electronic conduction through the M layer in an MXene. As such they can have conductivities reaching 20,000 S cm^–1^, making them excellent candidates for semitransparent electrical contacts.^16^ The work function of an MXene varies based on its transition metal M and the surface terminations of the flake, with work functions up to 8 eV predicted for some MXenes, well-beyond the high ionization potential of a CdTe surface, at 5.9 eV.^17,18^ Previous investigations have shown that Ti_3_C_2_T_*x*_, the prototypical and most studied MXene with a work function of 5.3 eV, is well-aligned with CdTe.^19^ Due to its tunable work function and high conductivity, Ti_3_C_2_T_*x*_ MXene has been explored in solar cell applications.^20,21^

Cracked film lithography (CFL), developed and applied by Muzzillo et al., is a rapid and low-cost method for fabricating metallic nanogrids by leveraging the spontaneous formation of crack networks in colloidal solutions as they dry. By depositing a metallic contact through the crack network and lifting off the nanoparticle film following deposition, we left an optically transmissive and electronically conductive back contact nanogrid on the surface. While carriers generated in the vicinity of the grid fingers are efficiently collected, there is significant resistance experienced by carriers generated away from the grid fingers, as they must conduct laterally through the highly resistive CdTe and AlGaO_*x*_ layers to reach the metallic contact. To maximize the benefit from the CFL nanogrid, additional lateral conductance across the CdTe surface is necessary. Given the high conductivity and ideal band alignment, Ti_3_C_2_T_*x*_ forms a low-barrier transparent contact to the AlGaO_*x*_ passivated CdTe surface. It also provides the required additional conductance to carry electrons into a transparent nanogrid contact templated by a CFL. In this work, we demonstrate this hierarchical approach that addresses energy band alignment, transparency, and series resistance in turn.

The hierarchical approach described in this work leverages several materials to address band alignment and series resistance (Figure 1d). The first layer, AlGaO_*x*_, has an ideal band alignment with the CdTe back surface, improving the open-circuit voltage. The second layer, Ti_3_C_2_T_*x*_ MXene, is used to form a transparent ohmic contact with the AlGaO_*x*_ buffer layer, reducing series resistance by improving micrometer-scale lateral conductivity. Finally, a cracked film lithography patterned gold nanogrid is installed on top of the MXene to increase macroscale in-plane conductivity while maintaining high transparency.

All of the devices presented use CdSeTe absorbers grown by First Solar. XPS analysis of neat AGO films shows that AlGaO_*x*_ is an amorphous oxynitride, containing residual nitrogen from nitrate salt precursors (Figure S1). As shown in Figure 2a, when gold contacts are deposited on top of AlGaO_*x*_ rather than a pristine absorber surface, open-circuit voltage (*V*_OC_) is boosted from 735 to 826 mV. Examining these devices by temperature-dependent current-density voltage (*JV*) measurements (Figure 2b) shows that the AlGaO_*x*_-treated samples have a lower rear contact barrier, reducing the barrier from 0.46 to 0.31 eV. Previous studies have demonstrated that a 0.15 eV shift in contact barrier can give a sizable shift in *V*_OC_ in the presence of a high rear surface recombination.^22^ In contrast to the results presented in Akkuly et al. on undoped CdTe absorbers, AlGaO_*x*_ does not affect the 1PE time-resolved photoluminescence (TRPL) decay in unmetallized arsenic-doped CdSeTe devices, suggesting surface passivation is unaffected (Figure 2c).^12^ Metallization results in a decreased τ_2_ lifetime as measured by TRPL, with a larger decrease in τ_2_ lifetime for the AlGaO_*x*_-treated sample, despite the latter resulting in a higher efficiency device. The beneficial impact of the back contact barrier reduction may outweigh any detrimentally reduced τ_2_ lifetime. A recent study demonstrated that in undoped CdSeTe, high τ_2_ lifetimes might correspond to trapping/detrapping mechanisms that not only increase τ_2_ but also impart a lower hole mobility on the absorber, increasing the sensitivity of *V*_OC_ to the magnitude of any back contact barriers.^23^ Despite the improved *V*_OC_ with continuous gold contacts, AlGaO_*x*_ cannot be used directly with CFL-templated nanogrids due to the high series resistance stemming from the low in-plane conductivity of the AlGaO_*x*_ layer. Au nanogrids deposited on this AlGaO_*x*_ layer resulted in similarly improved *V*_OC_ but a drastic reduction in fill-factor (70.2% to 41.4%) due to the introduction of additional series resistance (Figure 4a). This detrimental effect must be avoided to leverage the beneficial impacts of AlGaO_*x*_ and CFL templating for a bifacial device.

**Figure 2:**
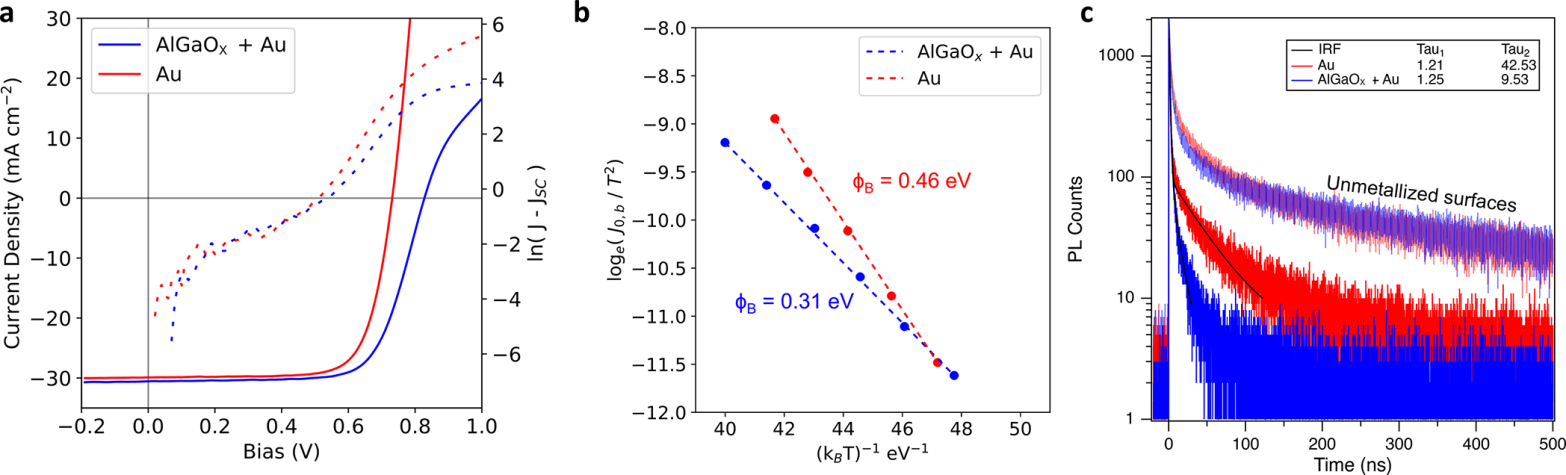
(a) *J–V* characteristics for a CdTe/gold and a CdTe/AlGaO_*x*_/gold device in both linear (solid) and semilogarithmic (dashes) plots. (b) An Arrhenius plot of the back contact saturation current yielding the rear contact barrier height for CdTe/Gold and CdTe/AlGaO_*x*_/gold devices. (c) TRPL measurements showing a reduced τ_2_ in the AlGaO_*x*_/gold contacted device.

An intermediary conductive layer may impart in-plane conductivity to shuttle generated carriers into the highly conductive nanogrid and improve the device fill factor (FF). By modeling a back contact composed of a Ti_3_C_2_T_*x*_ MXene thin-film and a CFL nanogrid, we find optimum thicknesses and grid spacings that will enable both a low series resistance and a high transmissivity. We assumed a simple illuminated diode in series with a resistor to model this system in Python. Optical parameters for Ti_3_C_2_T_*x*_ were reported by Fu et al. and used in this model.^24^ Determination of the series resistance of a CFL nanogrid in contact with a “neighbor” semiconductor or metal is described elsewhere.^25^ The model description can be found in the Supporting Information (S2), but it notably does not account for interfacial effects such as surface recombination and back-contact barriers. By modeling the power output of a device under one sun illumination through a transmissive contact and varying the Ti_3_C_2_T_*x*_ thickness and CFL grid spacing, an optimal device was found to have a 1 nm thick Ti_3_C_2_T_*x*_ film and a 20 μm grid spacing, with a 47% improvement in the power output over a device with an optimized CFL grid but no intermediary conductive layer (Figure 3a). This trend persists regardless of the device quality, as proxied by saturation current density *J*_0_ (Figure 3b). This result is a first-order guideline for optimizing a semitransparent contact, but an improved model incorporating spatially resolved carrier generation, nonequilibrium band bending at the back interface, and back surface recombination would more accurately account for losses in a bifacial device.

**Figure 3:**
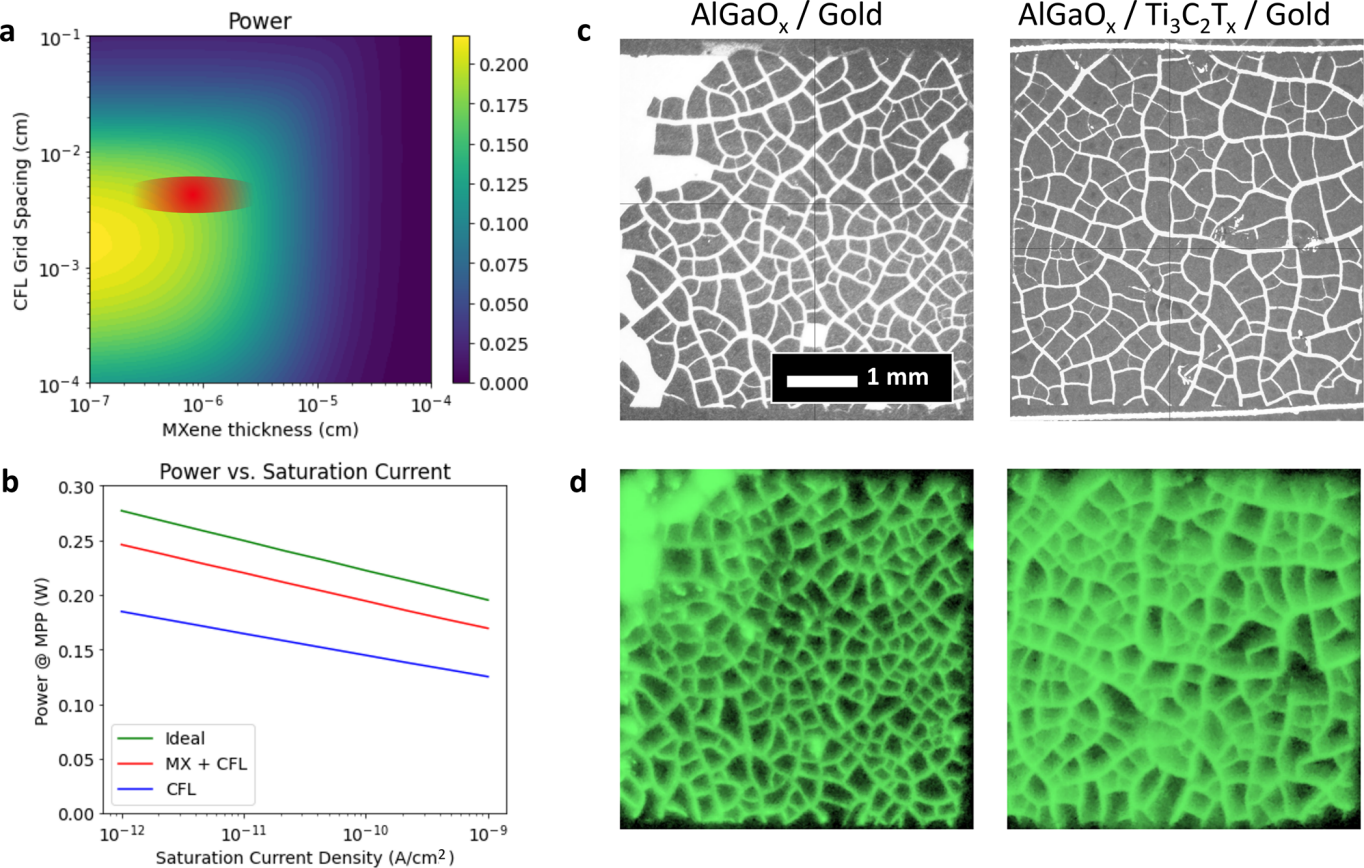
(a) Simulated backside power as a function of Ti_3_C_2_T_*x*_ thickness and CFL spacing. The red spot represents experimentally demonstrated grid spacing and deposited MXene thicknesses. (b) Comparison of combined Ti_3_C_2_T_*x*_ and CFL, and CFL-only devices with an idealized device as a function of device quality (proxied by saturation current density). (c) Optical microscopy images of CFL grids as measured from the film side of the device. (d) Electroluminescence imaging of the same CFL grids measured from the film side.

This proposed intermediary conductive and transparent layer must still satisfy the energy-band alignment to avoid the formation of a Schottky barrier, ruling out several potential transparent conductive oxides. High work-function (5.3 eV) Ti_3_C_2_T_*x*_ MXenes satisfy these requirements. A First Solar CdSeTe device stack was completed with an AlGaO_*x*_ passivation layer, a semitransparent Ti_3_C_2_T_*x*_ contact, and a 1.2 μm thick CFL nanogrid. Electroluminescence images were measured from the glass side of both a CFL-nanogrid only and a CFL-nanogrid with a Ti_3_C_2_T_*x*_ interlayer (Figure 3c,d). In the device containing the Ti_3_C_2_T_*x*_ interlayer, the luminescence spreads more uniformly into each “cell” of the nanogrid, indicating that the impact of high series resistance spots present in the CFL-only device at the center of each “cell” has been alleviated (Figure S3).

This hierarchical back contact greatly reduced the back contact barrier and improved the fill factor from 56.2% to 81.5%, yielding a 19.5% efficient device when measured from the frontside (Figure 4a, Table 1). PCE_Back_ was measured to be 2.9% under 1-sun illumination. In the devices in contact with Ti_3_C_2_T_*x*_, any rollover characteristic associated with a back contact barrier was eliminated from the device (Figure 4b). In comparison, a device using an indium zinc oxide (IZO) transparent conductive oxide as the intermediary conductive layer suffered from poor band alignment, introducing a significant contact barrier that eroded the device fill factor and efficiency. External quantum efficiency (EQE) measurements were conducted to investigate current losses and accurately calibrate the device area (Figure 4c). While the front-side QE was as expected, the back surface QE indicated a highly unpassivated back surface, with most of the current coming from the spectrum region associated with low-bandgap Cd(Se,Te) at the front interface. By leveraging the high work function and conductivity of a Ti_3_C_2_T_*x*_ intermediary contact layer, the ceiling on fill factor associated with series resistance and contact barriers from gold-only contacts was alleviated, improving upon the fill factor of the state-of-the-art device reported in Muzzillo et al. by 8%.

**Figure 4:**
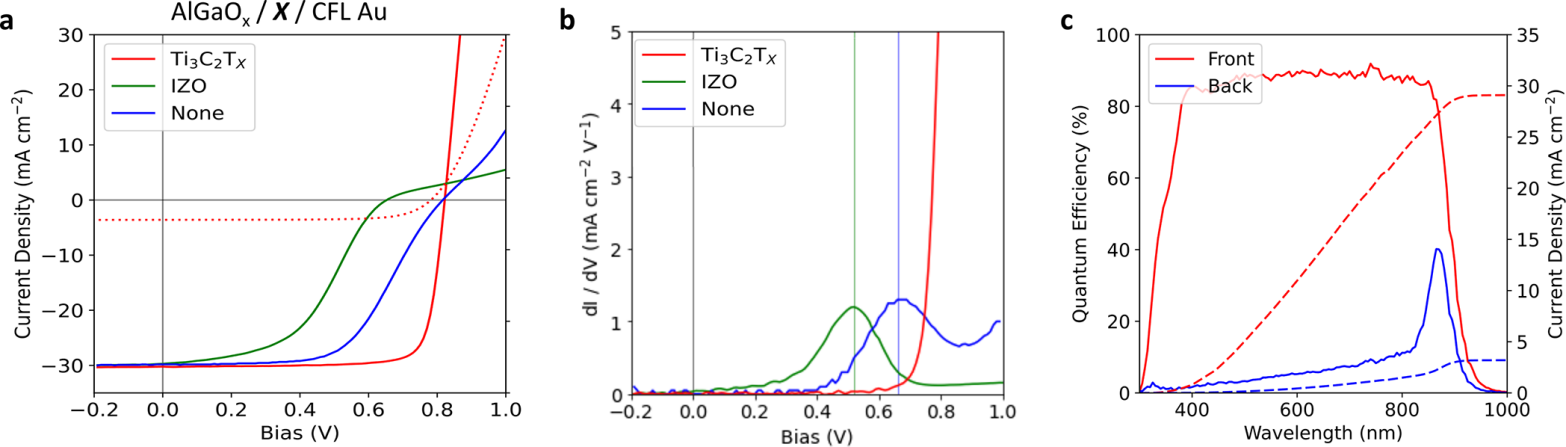
(a) *J–V* data for AlGaO_*x*_-passivated dot cells. CFL Au has a significant “roll-under”, limiting the FF. Introducing a Ti_3_C_2_T_*x*_ interlayer removes the FF issue, while using an alternative transparent conductor such as IZO does not. *J–V* under backside illumination is shown by a dashed line. (b) Derivative of *J–V* illustrating the onset of “kink” in a curve, indicating the presence of a back contact barrier in IZO/gold and gold-only contacted devices. (c) EQE for frontside and backside of AlGaO_*x*_-passivated, Ti_3_C_2_T_*x*_, and CFL aluminum contacted CdTe device. Integrated current density (dashed) was used for area correction in the *J–V* measurement. Low quantum efficiency up to 800 nm indicates poor back-surface passivation.

**Table 1:** *JV* Characteristics of Hierarchical Back Contacts Applied to Arsenic-Doped First Solar Absorbers^a^

**Contact**	**Fill Factor (%)**	**Short Circuit Current (mA/cm**^**3**^**)**	**Open Circuit Voltage (V)**	**Photoconversion Efficiency (%)**
Gold	75.0 ± 1.0	31.1 ± 0.5	735 ± 7	17.1 ± 0.7 (17.9)
AlGaO_*x*_ + Gold	70.5 ± 0.1	31.1 ± 1.0	826 ± 1	18.1 ± 0.5 (18.8)
AlGaO_*x*_ + CFL Gold	41.4 ± 3.6	19.0 ± 2.6	838 ± 6	6.7 ± 1.6 (10.2)
AlGaO_*x*_ + IZO + CFL Gold	46.4 ± 1.2	30.4 ± 1.6	646 ± 7	9.1 ± 0.7 (9.6)
**AlGaO**_***x***_**+ Ti**_**3**_**C**_**2**_**T**_***x***_**+ CFL Gold**	71.2 ± 4.9	30.3 ± 1.6	844 ± 13	18.1 ± 1.0 **(19.5)**
***Champion Device***	***81.5***	***29.1***	***821***	***19.5***
***Backside***	79.0 ± 2.4	4.0 ± 0.2	797 ± 7	2.6 ± 0.2 **(2.9)**
***Thin Absorber***	61.8 ± 4.4	29.5 ± 1.1	741 ± 29	13.5 ± 1.4 **(15.3)**
***Thin Absorber Backside***	76.7 ± 1.8	8.0 ± 0.7	743 ± 4	4.5 ± 0.4 **(4.9)**
CuGaO_*x*_ + CFL Gold Muzzillo (2023)	73.3	30.9	848	19.2
*Backside*	76.2	4.7	787	2.8

a Champion device efficiencies are provided inside the parentheses. The First Solar absorbers used by Muzzillo et al. were copper-doped.

These devices contain no intentional copper and thus are more closely aligned with the next generation of CdSeTe devices using arsenic dopant chemistry. The PCE_Back_ of 2.9% is notable in that it was achieved on an unoptimized First Solar substrate with a high carrier concentration from the arsenic dopant chemistry. Past results have benefited from larger depletion widths associated with Cu doping and the use of a thinner substrate, resulting in a larger percentage of carriers generated inside the depletion width of a device under backside illumination. By leveraging a thin (1.5 μm) group-V doped absorber fabricated by First Solar, we improved the backside efficiency to 4.9% but at the expense of frontside efficiency (Figure 5a,b). By thinning the CdSeTe between the depletion region and the back surface where light enters, a higher percentage of carriers is generated inside the depletion width and contributes to the current density. However, to the detriment of front-side efficiency, this shorter distance between the frontside and backside results in more interaction between the still imperfect back surface and the main junction, resulting in a reduced *V*_oc_ and FF.

**Figure 5:**
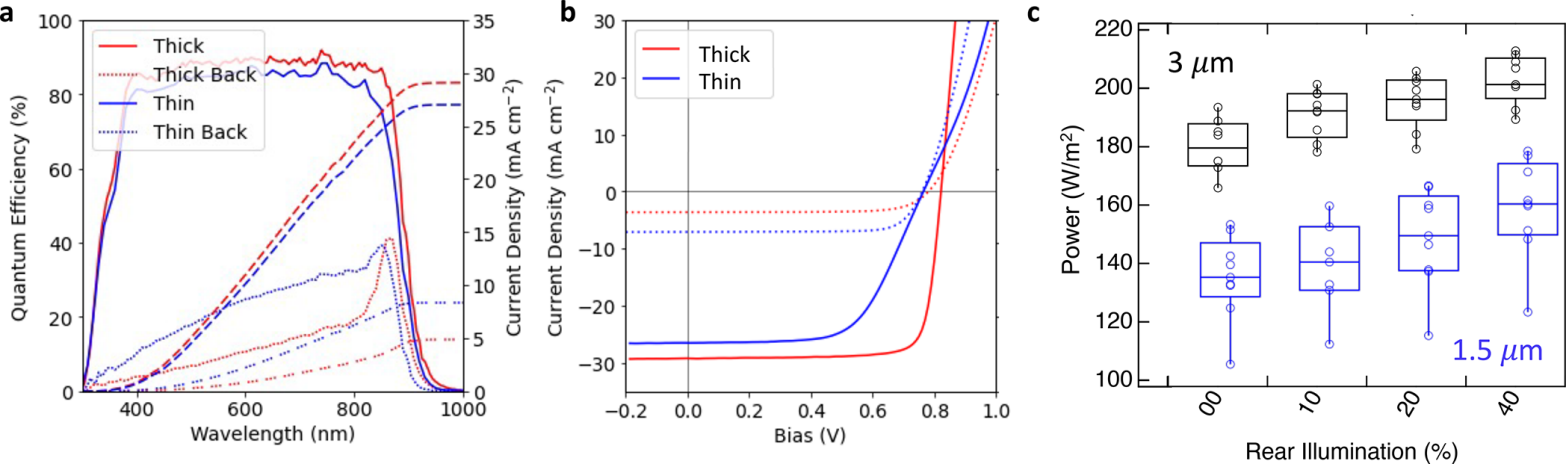
(a) Frontside (solid) and backside (dashed) EQE for both thin (blue) and thick (red) CdSeTe absorbers with AlGaO_*x*_/Ti_3_C_2_T_*x*_/CFL Au contacts. (b) *J–V* data for thick (red) and thin (blue) devices under frontside and backside illumination. (c) Power output for thin and thick devices under combined frontside and backside illumination with varied backside illumination.

Dual illumination with AM1.5G on the front surface and a percentage of AM1.5G on the back surface can provide a more field-accurate simulation of the bifacial performance, simulating reflected light from a surface underneath the cell with an arbitrary albedo. While estimates suggest that this backside illumination from reflection can reach up to 90%, such as when a panel is over snow, the maximum reflection will be closer to 50% under most conditions.^26^ Accounting for typical panel density in a field, the expected incident light on a back surface is approximately 10%. We demonstrate that with 10% AM1.5G illumination on the back surface, the device power generation surpasses 200 W/m^2^ (Figure 5c), the equivalent power generation to a 20% monofacial device under AM1.5G. This is from a slight increase in *J*_sc_, 30.8 to 31.1 mA/cm^2^, but an even more impactful increase in fill factor, 69.8% to 72.2%, beyond what could be accounted for by increased generation from more incident light (Figure S4). While it is expected that *J*_sc_ should drive power generation improvement in bifacial operation, we find that the fill-factor improvement is 4× more impactful.^27^ We hypothesize that this effect may be due to a filling of trap states near the back surface, reducing detrimental band-bending and recombination near the back interface and thus resistance near the max power point and *V*_OC_. However, this effect merits further research and remains beyond the scope of this letter. With 40% AM1.5G illumination on the backside during dual illumination, power generation reaches 213 W/m^2^.

AlGaO_*x*_ passivation improves the bifacial potential of a copper-free CdSeTe device by improving the *V*_OC_. To avoid the introduction of detrimental Schottky barriers or series resistances, a bilayer Ti_3_C_2_T_*x*_ and CFL-templated nanogrid were used to make a series of CdSeTe devices with a champion PCE of 19.5% and a PCE_Back_ of 2.9%. By leveraging thin absorbers, the *J*_SC_ under backside illumination was increased at the expense of frontside *V*_OC_ and FF. Under dual illumination, device fill factors improved significantly, even under the lowest illumination of 10% AM1.5G. The champion cell demonstrates a record combined front and backside efficiency for a bifacial CdSeTe cell and over 200 W/m^2^ under conservative bifacial operation with 10% AM1.5G backside illumination. These hierarchical back contacts outperform either cracked film lithography templated grids or thin Ti_3_C_2_T_*x*_ alone and demonstrate a strategy to address the challenges of bifacial contacts in a systematic way. This hierarchical approach is highly modular in its application, allowing for device improvements to be achieved as material properties improve for each layer, such as an improved passivation layer compared to AlGaO_*x*_ or higher transparency and conductivity MXenes. Additional layers to consider in such a hierarchical approach may include molecular-scale passivation layers at the rear surface, which have seen tremendous success in passivating the surfaces in thin-film perovskites,^28,29^ or optical layers behind the CFL grid for long-wavelength light management. As the hierarchical approach presented is a strategy to manage series resistance, transmissivity, and contact barrier height effectively, it enables competitive bifacialities when it is used in tandem with these additional layers. For example, Phillips et al. simulate a bifaciality increase from 16% to 67% with a reduction in back surface recombination velocity from 10^3^ to 10^2^ cm/s.^27^ The latter would be achievable with state-of-the-art Al_2_O_3_ passivation over 90% of the area using our contact structure.^30^ Moreover, combining arsenic doping with low back surface recombination velocity would enable the use of thinner absorbers, increasing carrier generation near the front junction field to achieve upward of 90% bifaciality.^4^

## Supporting Information Available

Experimental methods, XPS data for AlGaO_x_ layers, Python model description for simulating performance of CFL nanogrid and Ti_3_C_2_T_*x*_ MXene contacts; Quantitative treatment of CFL nanogrid electroluminescence maps; Full device photovoltaic characteristics under varied dual-illumination intensities (PDF)
