# Supplementary material for: Hierarchical Transparent Back Contacts for Bifacial CdTe PV
Source: ACS Energy Lett. 2024 Mar 20;9(4):1617–23. doi: 10.1021/acsenergylett.4c00156 (PMC11019634; doi:10.1021/acsenergylett.4c00156)
Supplement: Supplementary file 1 — nz4c00156_si_001.pdf [file nz4c00156_si_001.pdf]

Hierarchical transparent back contacts for bifacial CdTe PV

B. Edward Sartor[1,2], Teng Zhang[3], Christopher P. Muzzillo[2], Chungho Lee[4], Ryan Muzzio[2], Yury Gogotsi[3], Matthew O. Reese[2], Andre D. Taylor[1]

1. New York University, Brooklyn, New York, 11201, USA
2. National Renewable Energy Lab, Golden, Colorado, 80401, USA
3. Drexel University, Philadelphia, Pennsylvania, 19104, USA
4. First Solar, Santa Clara, California, 95050, USA

Corresponding Author

Andre D. Taylor

adt4@nyu.edu; 6 Metrotech Center, Brooklyn; 11201 NY

First Author
B. Edward Sartor
ed.sartor@nrel.gov; 15013 Denver West Parkway, Golden; 80401 CO

Experimental methods

Partially completed arsenic-doped Cd(Se)Te devices were provided by First Solar. $Ti_3C_2T_x$ MXenes were prepared at Drexel University by the "mixed acid" method, described elsewhere,[1] and then diluted to 5 mg $Ti_3C_2T_x$ per mL H_2O immediately before use. TiO_2(IV) nanopowder (CAS: 1317-80-2), $Al(NO_3)_3 \bullet 9H_2O$ (CAS: 7784-27-2), $Ga(NO_3)_3 \bullet xH_2O$, N,N-dimethylformamide (DMF), dimethylsulfoxide (DMSO), and ethanol were purchased from Millipore Sigma. $AlGaO_x$ precursor solutions were prepared by adding 187 mg of $Al(NO_3)_3 \bullet 9H_2O$ and 77 mg of $Ga(NO_3)_3 \bullet xH_2O$ to 10 mL of DMF. Cracked film lithography templates were synthesized by adding 11 wt% TiO_2 to a 10:1 ethanol-DMSO mixture.

First Solar absorbers were washed with DI water before $AlGaO_x$ deposition. $AlGaO_x$ was deposited via spin-coating at 1000 rpm for 10 seconds and 2500 rpm for 50 seconds, followed by a 220°C hotplate anneal for 20 minutes in ambient air. $Ti_3C_2T_x$ was deposited on top of the $AlGaO_x$ layer by spin-coating under the same spin rates and then allowed to dry in the air for several minutes. The cracked film lithography template was deposited by drop-casting 180 µL of TiO_2 solution / in^2 of the sample, then allowed to dry overnight. The devices were then loaded into an Angstrom thermal evaporation system where 100 nm of 7 N gold was thermally evaporated onto the surface, followed by 100 nm of nickel and 1 **µ**m of aluminum without breaking the vacuum. The template was then gently washed by swirling the devices in deionized H_2O. In the samples with IZO rather than $Ti_3C_2T_x$ as the second deposited layer, 50 nm IZO was sputtered onto the surface.

J-V, *J-V*(*T*), TRPL,[2] and EL were measured on instruments designed and built at NREL. EQE was measured on a Newport Oriel IQE-200.

Supporting information

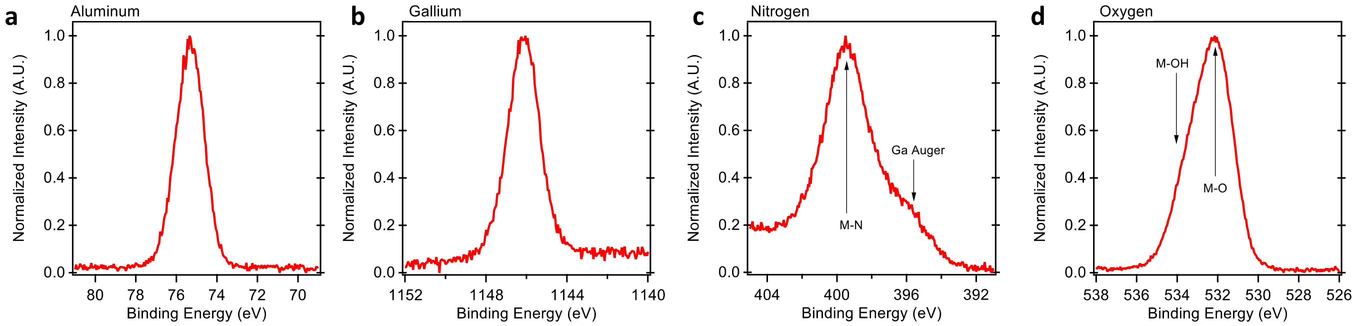

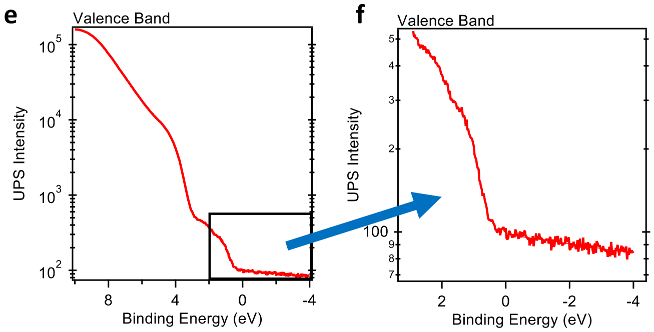

g

Element	Atomic %
Al	17
Ga	8
O	56
N	19

Figure. S1. a) Aluminum 2p core level. b) Gallium 2p core level. c) Nitrogen 1s core level, with shouldering for Ga Auger peak d) Oxygen 1s core level. e) Valence band edge f) Magnified valence band edge at Fermi-level. g) Atomic concentrations from XPS.

S2. Python modeling of cracked-film lithography templated metal and MXenes

$$T_{MX} = (1 + 188.5\,\sigma_{OP} t_{MX})^{-2}$$

$$T_{CFL} = \frac{s}{s + w}$$

$$T = T_{MX} \times T_{CFL}$$

$$I_L = T \times I_{in}$$

The total transmission through the contact is calculated from the product of transmission through the MXene thin film and the non-uniform cracked film lithography-templated (CFL) microgrid, modeled here as a cell with the distance between grid fingers “s” and grid finger width “w”. The grid finger width is fixed at 100 μm and the distance between grid fingers is varied to find a maximum power. The current “I_L” is the input current “I_{in}” attenuated by the transmissivity of the combined CFL microgrid and MXene film.

$$R_{\square} = \sigma_{DC,MX}\, t_{MX}$$

$$R_{MX} = R_{\square} J\, s^2$$

$$R_{CFL} = \frac{sL^2}{3\sigma_{DC,Al}(t_{Al} * (w))}$$

$$R = R_{S,MX} + R_{S,CFL}$$

The total series resistance introduced by the combined CFL microgrid and MXene film is calculated as the sum of the individual resistances contributed by each layer. The sheet resistance of the MXene film is calculated from the reported conductivity of $Ti_3C_2T_x$ and the thickness.[4] The CFL-microgrid contribution to series resistance is calculated following the procedure for estimating series resistances of metal grids in contact with a neighbor semiconductor in Muzzillo *et al.*[5]

$$I = I_L - I_0 \exp\left(\frac{q(V + IR)}{nkT}\right)$$

The ideal diode equation (with a non-ideality factor of 1.5) is used to calculate the maximum power point given varied MXene layer thickness and CFL microgrid finger spacing by leveraging the estimated light-induced current I_L and resistance *R*. These maximum power points are shown in Figure 3a. In Figure 3b, the maximum power point is found for each CFL microgrid finger spacing for the case of an arbitrarily thick MXene layer with CFL microgrid, a CFL microgrid only, and a “perfect” contact with no series resistance or transmission losses.

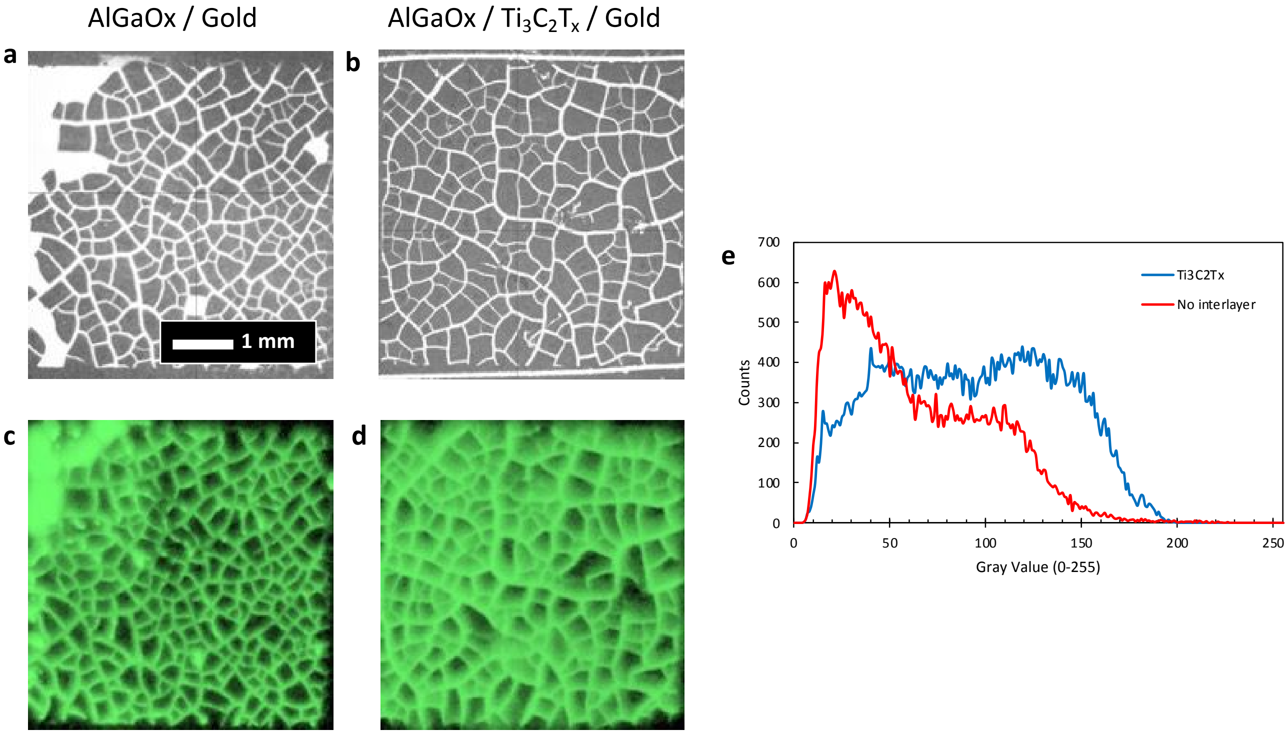

Figure. S3. a,b) Optical microscopy images of CFL grids as measured from the film-side of the device. C,d) Electroluminescence (EL) imaging of the same CFL grids measured from the film-side. E) EL images integrated over the entire image to bin grey-values from the detector. Higher intensity emission corresponds to a higher grey value. As MXenes are applied, the density of grey-values shifts from lower values to higher values for the same voltage and exposure time, indicating that EL emission intensity is more uniform and high across the device.

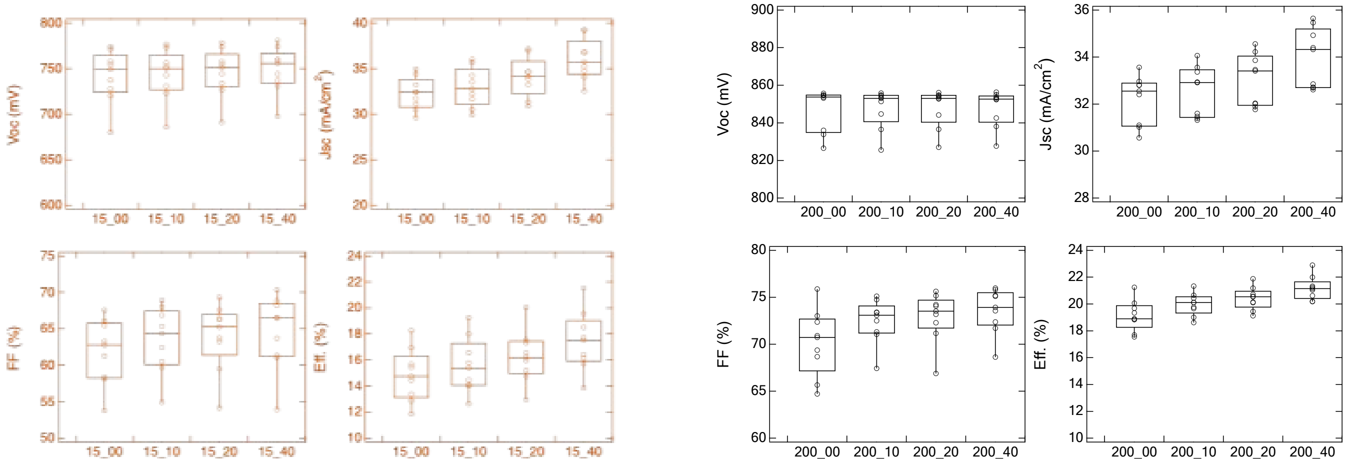

Figure. S4. V_{OC}**,** J_{sc}**, FF, and PCE for 1.5 µm thick (left) and 3 µm thick (right) CdSeTe devices with a CFL microgrid + MXene contact with 1 sun frontside illumination and 0, 0.1, 0.2, 0.4 sun backside illumination. PCE is relative to 1 sun efficiency, but incident illumination is higher due to dual illumination, thus power generation in W/m^2 is reported in the manuscript instead of PCE.**

References:

(1) Mathis, T. S.; Maleski, K.; Goad, A.; Sarycheva, A.; Anayee, M.; Foucher, A. C.; Hantanasirisakul, K.; Shuck, C. E.; Stach, E. A.; Gogotsi, Y. Modified MAX Phase Synthesis for Environmentally Stable and Highly Conductive Ti_3C_2 MXene. *ACS Nano* **2021**, *15* (4), 6420–6429. https://doi.org/10.1021/acsnano.0c08357.

(2) McGott, D. L.; Good, B.; Fluegel, B.; Duenow, J. N.; Wolden, C. A.; Reese, M. O. Dual-Wavelength Time-Resolved Photoluminescence Study of $CdSe_xTe_{1-x}$ Surface Passivation via $Mg_yZn_{1-y}O$ and Al_2O_3. *IEEE Journal of Photovoltaics* **2022**, *12* (1), 309–315. https://doi.org/10.1109/JPHOTOV.2021.3124169.

(3) Fox, J. R.; Kuciauskas, D.; Albin, D. S.; Nardone, M. Numerical Analysis of Time Resolved Photoluminescence for Alumina/Cd(Se,Te) Double Heterostructures. In *2021 IEEE 48th Photovoltaic Specialists Conference (PVSC)*; 2021; pp 0551–0556. https://doi.org/10.1109/PVSC43889.2021.9518978.

(4) Fu, B.; Sun, J.; Wang, C.; Shang, C.; Xu, L.; Li, J.; Zhang, H. MXenes: Synthesis, Optical Properties, and Applications in Ultrafast Photonics. *Small* **2021**, *17* (11), 2006054. https://doi.org/10.1002/smll.202006054.

(5) Muzzillo, C. P. Metal Nano-Grids for Transparent Conduction in Solar Cells. *Solar Energy Materials and Solar Cells* **2017**, *169*, 68–77. https://doi.org/10.1016/j.solmat.2017.04.048.
